# Long-term survival and prognostic factors in extragonadal germ cell tumors: a 30-year experience at a single institution

**DOI:** 10.1007/s12094-025-03904-2

**Published:** 2025-04-30

**Authors:** Patricia Capdevila, Cristobal Carrasco, Elisa Gómez, Carlos Escrivá, Emilio Soria, Josep M. Esteve, Jorge Aparicio Urtasun

**Affiliations:** Department of Medical Oncology, Hospital Universitario y Politécnico La Fe, Avda. Abril Martorell 106, 46016 Valencia, Spain

**Keywords:** Germ cell cancer, Extragonadal tumors, Mediastinal non-seminoma, Prognosis, Chemotherapy

## Abstract

**Purpose:**

Extragonadal germ cell tumors (EGCT) are rare malignancies, typically located in midline structures, with treatment consisting of cisplatin-based chemotherapy. We aimed to evaluate long-term outcomes, identify prognostic factors and assess treatment efficacy.

**Methods:**

We retrospectively evaluated 31 patients with EGCT between 1994 and 2024. RStudio was used for data analysis. Progression free survival (PFS) and overall survival (OS) was assessed using Kaplan–Meier, and prognostic factors via Cox regression. Approval was obtained from our local Ethics Committee.

**Results:**

The most common site was mediastinum (59%), followed by retroperitoneum and central nervous system. Non-seminomatous germ cell tumors (NSGCT) were more frequent than seminomas (61% vs. 39%), and 41% of patients had metastasis at diagnosis. After first-line chemotherapy plus selective surgery or irradiation, the disease control rate was 81%. Progression or relapse occurred in 48% of patients, mostly in those with mediastinal NSGCT. No secondary malignancies were detected during follow-up. 5- and 10-year OS were 70%. NSGCT histology and mediastinal location were significantly associated with lower survival, with a 5-year OS of 34% in mediastinal NSGCT.

**Conclusions:**

While cisplatin-based chemotherapy remains effective for EGCT patients, mediastinal NSGCT pose significant challenges, highlighting the need for improved strategies.

## Introduction

Extragonadal germ cell tumors (EGCT) represent a rare subset of germ cell tumors (GCT), accounting for 1–5% of all GCTs. Unlike gonadal tumors, EGCT arise in midline locations, most commonly in the mediastinum, retroperitoneum, and central nervous system, without detectable primary tumors in the testes or ovaries [1]. The mechanisms behind EGCT development are unclear, though theories suggest aberrant migration of primordial germ cells during embryogenesis [2]. Primordial germ cells (GC) originating from the proximal epiblast normally migrate to the genital ridge following the body midline. The thymus could be a preferential site for primordial GC arrest, because of high expression of KIT ligands, involved in primordial GC proliferation [3]. Although this theory is widely accepted, some authors proposed alternative etiopathogenetic explanations. McKenney et al. argued that EGCTs could represent metastases developed from undiagnosed or regressed (“burned out”) primary GC tumors in the gonads [4].

While advancements in cisplatin-based chemotherapy have significantly improved outcomes for patients with GCT, the prognosis for those with EGCT varies widely depending on tumor histology, location, and metastases. Mediastinal NSGCTs have particularly poor survival [5]. In addition, EGCT often present at an advanced stage, complicating management and highlighting the need for more effective therapeutic strategies. Elective treatment consists of cisplatin-based chemotherapy plus judicious use of surgery or radiation therapy for residual lesions. Tumor-marker decline is a cornerstone in monitoring systemic treatment [6]. Finally, these patients are recommended to be treated in high-volume referral centers [7].

Given the rarity of EGCT, large-scale studies are limited, and clinical data are often derived from small case series. In this study, we aimed to assess the long-term outcomes of EGCT patients treated at a single center. Our objectives were to evaluate survival rates, identify prognostic factors influencing outcomes, and assess the efficacy of current treatment approaches.

## Methods

A retrospective evaluation was conducted on 343 patients diagnosed with GCT and prospectively registered at our center between 1994 and 2024. EGCT was defined by germ-cell histologic confirmation and absence of testicular abnormalities. Clinical and demographic data were extracted from medical records. Detailed information was acquired on patient characteristics, such as location and histology of primary tumor, raised serum markers (above upper limit); diagnostic methods, treatment, and response; follow-up period and data on second cancers. Duration of follow-up and survival were calculated from the date of diagnosis until the date of last contact or death.

Disease control rate (DCR) was assessed in all patients and was defined as the absence of evidence of progression at the follow-up disease assessment and comprended the sum of partial (PR), complete response (CR) and stable disease (SD). Disease-specific survival (DSS) was calculated as the probability of survival, censoring noncancer causes of death. Progression-free survival (PFS) was defined as the time to the detection of progressive disease. The Overall Survival (OS) calculation used death caused by any reason as the end point.

RStudio was used for data analysis. The Kaplan–Meier method was used to determine OS and PFS distributions. Clinical variables such as histology, location, raised serum markers, metastases at diagnosis, treatment used, response to chemotherapy and relapses were included into univariate analysis to identify their influence on PFS and OS. Comparison of resulting curves and univariate analysis of prognostic factors were carried out with the log-rank test. Statistical significance was set at a *p* value < 0.05.

## Results

A total of 343 patients with GCT were diagnosed at our center between 1994 and 2024, 31 (9%) presented EGCT. Median age was 31 years (range, 15–69 years). No story of cryptorchidism or Klinefelter syndrome was reported. The most common location was the mediastinum (61%), followed by retroperitoneum (23%) and central nervous system (CNS, 16%). Non-seminomatous histology was more frequent (61%), and 42% had metastases at diagnosis. Raised serum markers -AFP, β-hCG, and LDH- were observed in 42%, 48% and 58% of the patients, respectively. Patients with mediastinal GCT presented in 74% with non-seminomatous histology and in 58% with metastatic disease (Table 1).

**Table 1:** Demographic and clinical and pathological characteristics of extragonadal cancer patients

	Seminoma *n* (%)	TGNS *n* (%)	Total *n* (%)
*N* (%)	12 (38.7)	19 (61.2)	31 (100)
Median age (years, range)	34 (15–69)	27 (17–48)	31 (15–69)
Location			
Mediastinum	5 (41.7)	14 (73.7)	19 (61.3)
Retroperitoneum	4 (33.3)	3 (15.8)	7 (22.6)
Central nervous system	3 (25)	2 (10.5)	5 (16.1)
Pineal gland	2 (66.7)	2 (100)	4 (80)
Mesencephalon	1 (33.3)	0 (0)	1 (20)
Histology			
Embryonal carcinoma		6 (31.6)	6 (31.6)
Yolk sac tumor		3 (15.8)	3 (15.8)
Raised serum markers			
AFP +	0 (0)	13 (92.9)	13 (54.2)
BHCG +	7 (70)	8 (66.7)	15 (62.5)
LDH +	7 (87.5)	11 (91.7)	18 (90)
Metastasis at diagnosis			
Yes	4 (33.3)	9 (47.3)	13 (41.9)
No	8 (66.7)	10 (52.6)	18 (58.1)
Location of metastasis			
Ganglionar	1 (25)	2 (22.2)	3 (23)
Pulmonary	1 (25)	3 (33.3)	4 (30.8)
Non-pulmonary visceral	2 (50)	4 (44.4)	6 (41.2)

All patients received cisplatin-based chemotherapy. The most common regimens were bleomycin, etoposide, and cisplatin (BEP, *n* = 20), etoposide and cisplatin (EP, *n* = 7), and etoposide, ifosfamide, and cisplatin (VIP, *n* = 2). Six patients (19%) received consolidation radiotherapy, primarily for CNS tumors. Surgical resection of residual lesions was performed in 7 out of 17 patients (41%) showing a partial response (PR) to chemotherapy. Patients not undergoing surgery included CNS tumors (*n* = 5), those achieving a complete response after chemotherapy (*n* = 3), patients with major partial response and negative markers (*n* = 8), those who relapsed during or after chemotherapy (*n* = 7) and inoperable tumors (*n* = 1). Out of the patients who underwent surgery, 3 achieved pathologic complete response (Table 2).

**Table 2:** Treatment modalities used

	Seminoma *n* (%)	TGNS *n* (%)	Total *n* (%)
*N* (%)	12 (38.7)	19 (61.2)	31 (100)
Chemotherapy			
BEP	6 (50)	14 (82.3)	20 (69)
EP	6 (50)	1 (58.8)	7 (24.1)
TIP	0 (0)	2 (11.8)	2 (6.9)
Local treatment			
Surgery	1 (8.3)	6 (31.6)	7 (22.6)
Radiotherapy	4 (33.3)	2 (10.5)	6 (19.4)
No local treatment	7 (22.6)	11 (57.9)	18 (58)
Pathological response after surgery			
Complete pathological response	1 (100)	2 (33.3)	3 (42.9)
Viable tumor	0 (0)	4 (66.7)	4 (57.1)
Disease control rate			
Complete response	5 (41.7)	3 (15.8)	8 (25.8)
Partial response	7 (58.3)	10 (52.6)	17 (54.8)
Stable disease	0 (0)	1 (5.2)	1 (3.2)
No response	0 (0)	5 (26.3)	5 (16.1)
Relapse			
Yes	1 (8.3)	14 (73.7)	12 (38.7)
No	11 (91.7)	5 (26.3)	19 (61.3)

The overall disease control rate after chemotherapy was 83%, comprising 27% complete responses and 57% partial responses with negative tumor markers. Fifteen patients experienced disease progression on first-line chemotherapy or early relapse, 10 (66%) of which were mediastinal EGCTs and 14 (93%) having NSGCT histology. Salvage chemotherapy was administered to 12 patients, with regimens including cisplatin, ifosfamide, and paclitaxel (TIP, *n* = 10) or carboplatin/oxaliplatin combined with gemcitabine, with or without paclitaxel (*n* = 2). In addition, two patients received radiotherapy, two underwent surgery, and one underwent both surgery and radiotherapy. Following salvage treatment, five patients remain disease-free, seven experienced a second relapse, and three patients died. For relapsed patients, third-line treatment consisted of high-dose chemotherapy (HDCT) based on carboplatin and etoposide, with or without ifosfamide or cyclophosphamide, followed by autologous hematopoietic progenitor transplantation (*n* = 5). Two additional patients (14%) received further chemotherapy with TIP or oxaliplatin/gemcitabine, with one patient remaining free of disease at last follow-up.

After a median follow-up of 58 months, nine patients (29%) have died, all of whom had NSGCT. In addition, one patient with a mediastinal NSGCT died from a secondary hematologic malignancy. The OS rates at 5 and 10 years were 70%, while disease-specific survival rates at the same intervals were 73%. Notably, the 5-year survival rate for patients with EGCT exhibited considerable variability: 34% for those with mediastinal NSGCT compared to 100% for patients with seminoma. No late germ-cell relapses were detected, and no patient developed a metachronous testicular cancer during follow-up.

**Fig. 1:**
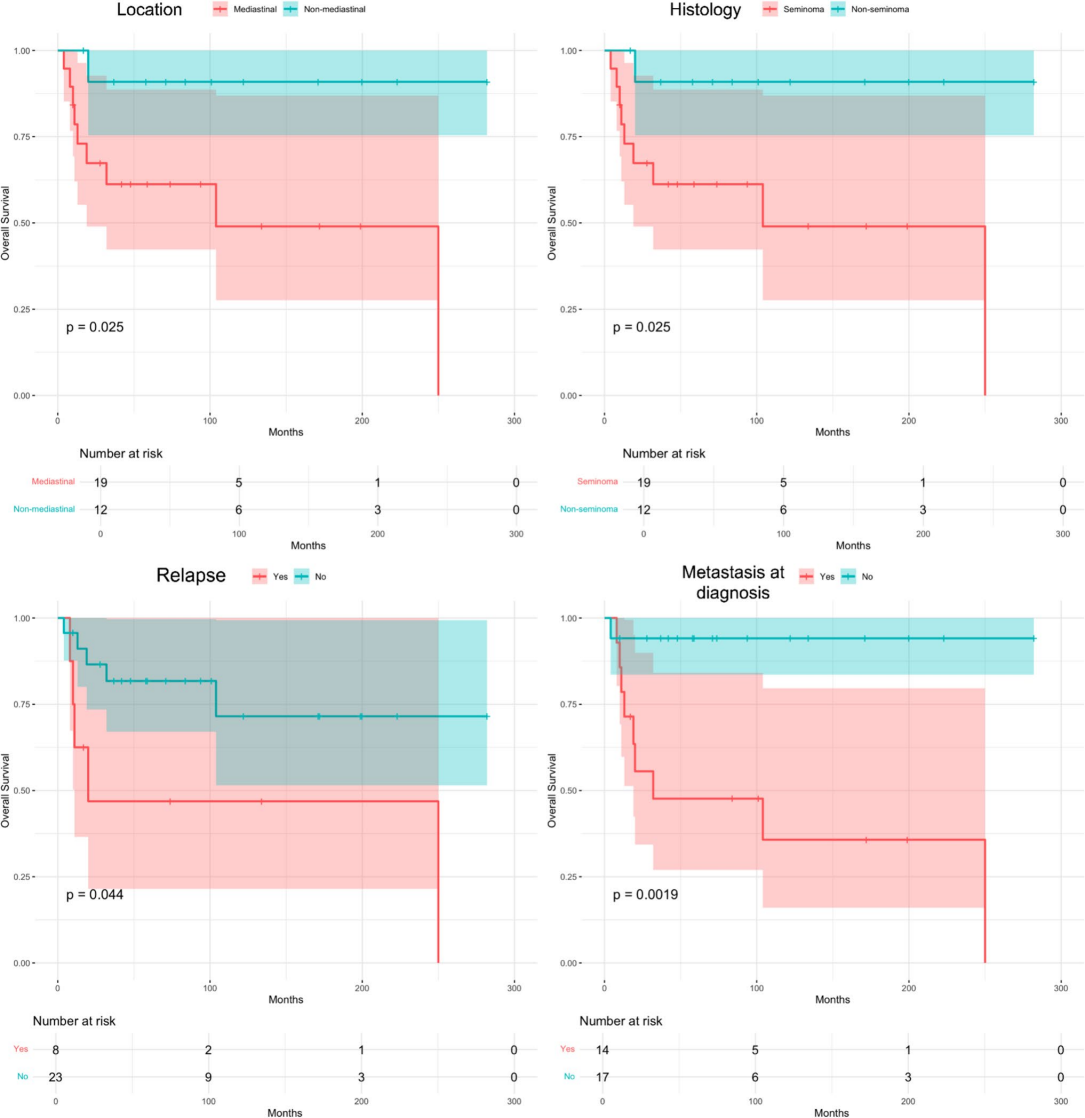
OS was significantly lower in patients with mediastinal tumors (*p* = 0.025), non-seminomatous histology (*p* = 0.025), those presenting relapse (*p* = 0.044) and those with metastasis at diagnosis (*p* = 0.0019)

Statistical analysis revealed that OS was significantly lower in patients with metastases at diagnosis (*p* = 0.006), NSGCT histology (*p* = 0.006), mediastinal primary (*p* = 0.003), poor response to chemotherapy (*p* = 0.001) and tumor relapse (*p* = 0.005) (Fig. 1).

## Discussion

This study provides a comprehensive analysis of EGCT, highlighting detailed long-term survival outcomes, prognostic factors, and treatment efficacy over three decades at a single center. The published literature is dominated by studies on testicular GCT, while there is limited population-based information on GCTs located at extragonadal sites. Despite being usually regarded as a single disease entity, they have considerable clinical and prognostic heterogeneity by tumor site and histology.

We retrospectively reviewed 31 cases of EGCT in a single Spanish institution. The median age was 31 years and 62% were NSGCT, features that are in line with previous reports [5, 8, 9]. The most common site in our series was the mediastinum (60%), in consonance with former large reports [1, 5, 10]. However, it contrasts with a 30-year experience in England, where the CNS was the most common extragonadal site [11]. Specifical hospital patient referrals could explain these differences. Thirteen patients (42%) presented metastatic disease at diagnosis, in 6 of them being extrapulmonary. Those with mediastinal location showed metastatic disease in 74%, a higher proportion compared to a Spanish report on mediastinal NSGCT (42% with metastases) [12]. High clinical suspicion, determination of serum markers (AFP and β-hCG) and histologic confirmation should guide the management of patients with mediastinal, retroperitoneal, and midline CNS tumors, in the setting of multidisciplinary tumor boards, to identify a subset of highly curable patients [6].

Cisplatin-based chemotherapy is the standard of care. In the current study, 16 of 31 patients treated with standard-dose cisplatin regimens, with or without secondary surgery, achieved a complete response and are alive after a median follow-up of 58 months: 7 mediastinal (41%), 5 retroperitoneal (71%) and 4 CNS tumors (80%). Our results are similar to previous series in terms of response and survival. In a Spanish report, 11 of 27 patients (41%) with mediastinal NSGCT achieved NED status (no evidence of disease) and 10 (37%) were alive with a median follow-up of 77 months [12]. Takeda et al. reported 5 out of 21 patients (24%) with NSGCT remained disease free with a median follow-up of 58.3 months and 10 of 13 patients (83%) with seminoma were alive and disease free [9]. Bokemeyer et al. described that 49% with mediastinal nonseminomas and 63% with retroperitoneal nonseminomas were alive after first line chemotherapy [5]. As reported by Hidalgo et al. [12], about 50% of patients relapsed (93% presented non seminoma histology in our series), and 30–35% of patients with relapsing or refractory EGCT are currently being cured with salvage treatment. Similarly, poor results in the salvage setting have consistently been reported by other groups, including those using HDCT [5, 8, 9]. In a recent report, 32 patients with mediastinal NSGCT were treated in the second or third line with HDCT and tandem peripheral stem cell transplantation at Indiana University. Their institutional approach includes also to attempt salvage surgical resection, either at first relapse or post-HDCT. Two-year OS was 35%, and 28% patients remained disease-free at last follow-up [13]. The authors recommend this approach for initial salvage therapy in this patient population.

Primary mediastinal origin has been identified as an adverse prognostic factor, probably related to an intrinsic resistance to cisplatin-based chemotherapy. However, these results should be interpreted with caution due to the small number and heterogeneity of the population which reduces methodological quality. Other poor indicators of outcome in this report are NSGCT histology, metastases at diagnosis, and relapses, which reproduce those reported in the IGCCCG classification [14]. These prognostic factors have also been reported by Bujanda et al. in a recent large population-based study [15]. Personalised (selective, dose-dense) chemotherapy based on tumour marker decline is a promising alternative therapy in poor prognosis germ-cell tumours based on GETUG 13, a phase 3, multicentre, randomised trial with 263 patients including 66 cases of primary mediastinal NSGCTs [16].

This study presents limitations. The small number of cases may affect the generalizability of the findings, as the study is based on a single institution's experience. The rarity of EGCTs contributes to the challenge of collecting a larger cohort, which is necessary for more robust statistical analysis and to draw more definitive conclusions about the clinicopathological features and treatment outcomes of these tumours. A collaborative effort within a national or international multicentric study should be carried out to generate more robust conclusions.

In conclusion, EGCT is a rare, heterogeneous, and potentially curable group of diseases. Despite therapeutic improvements in the past decades, the prognosis of patients with mediastinal NSGCT remains unsatisfactory. In this setting, future efforts should be directed at considering innovative first-line treatment approaches, attempting post-chemotherapy or salvage surgical resection, and considering HDCT as initial second-line therapy. All these procedures are better performed by multidisciplinary teams at highly experienced, referral centers.

## Data Availability

Authors are responsible for correctness of the statements provided in the manuscript.
